# Subanesthetic xenon increases erythropoietin levels in humans and remains traceable in the first 24 hours after exposure: a randomized controlled trial

**DOI:** 10.1186/cc14571

**Published:** 2015-03-16

**Authors:** J Ney, C Stoppe, M Brenke, A Goetzenich, S Kraemer, G Schaelte, A Fahlenkamp, R Rossaint, M Coburn

**Affiliations:** 1 RWTH Aachen University, Aachen, Germany

## Introduction

The noble gas xenon was recently amended to the list of prohibited substances by the World Anti-Doping Agency as it is supposed to trigger the production of HIF-1α and subsequently erythropoietin. Subsequently, researchers and clinicians started a scientific discussion about the potential clinical benefit in support of humans exposed to high demand, such as critically ill patients. The objective of this study was therefore to evaluate the effect of xenon on serum levels of erythropoietin in healthy volunteers.

## Methods

This is a monocenter, randomized, blinded, crossover trial, which was registered at http://ClinicalTrials.gov (NCT01285271). Healthy study test persons were spontaneously breathing randomly 1 hour of xenon 30% (Xe/O_2_ 30%/65%) or control gas (N_2_/O_2_ 30%/65%). The primary outcome parameter was the erythropoietin level 24 hours after exposure. Secondary outcome parameters are xenon's elimination kinetics measured in blood and exhalation samples.

## Results

**Figure 1:**
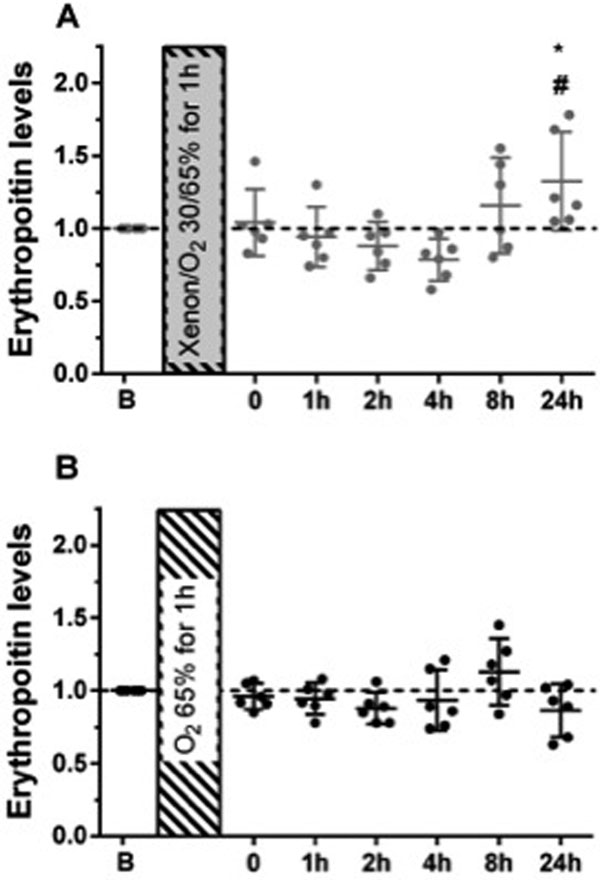
**Erythropoietin levels after 30% xenon exposure**.

The application of xenon increases erythropoietin levels with a maximum 24 hours after exposure (1.32 (0.99 to 1.66) *P *= 0.033) compared with the baseline values and compared with control values (0.87 (0.68 to 1.05) *P *= 0.012, Figure 1). Xenon was gas chromatographically traceable in blood and exhalation probes up to 24 hours after exposure.

## Conclusion

One hour of a subanesthetic level of xenon increases erythropoietin levels in healthy study test persons and remains gas chromatographically traceable in blood and exhalation probes 24 hours after exposure. These findings may stimulate larger studies to confirm these Results and to open new avenues for the therapeutic use of xenon in critically ill patients.

